# Traumatic Separation of the Lower Epiphysis of the Humerus

**Published:** 1903-12-12

**Authors:** 


### TRAUMATIC SEPARATION OF THE LOWER EPIPHYSIS OF THE HUMERUS.

There are few injuries which are fraught with more danger to the reputation of the medical man who is called upon to treat them than fractures. Those in the neighbourhood of the elbow joint are on exception. They are often hard to diagnose and are always difficult to manage with success and credit.

Separation of the lower epiphysis of the humerus is a particular form of this class of injury, which is not extremely rare, and which it is of the highest importance to recognise and deal with properly. In a paper on the subject, Dr. G. G. Cottam [1] gives the following points as being helpful towards making a correct diagnosis :—(1) Early and extensive swelling ; (2) preternatural mobility ; (3) deformity, which is easily overcome, but readily reproduced ; (4) muffled crepitus ; (5) shortening of the arm ; (6) limitation of active movements by pain ; (7) absence of resistance on flexing the forearm beyond a right angle with the arm. In addition to these there are, of course, the history of the accident, the age of the child, and the location of the injury. With regard to treatment, it is recommended by most authorities that the forearm should be retained at a right angle after the displacement has been rectified, and various reasons are put forward in support of this method. Dr. Cottam, however, is of opinion that the better plan is to keep the forearm in the position of full extension during the time that repair is taking place. He argues that if the forearm be kept flexed, the formation of callus during the process of repair will in part occupy the olecranon fossa, and so prevent subsequently the power of full extension. He has treated two cases of separation of the lower epiphysis of the humerus in this way with a perfectly good result in each instance.

[1] Medical Record, Nov. 14.

